# Bibliographical Notices

**Published:** 1847-09

**Authors:** 


### BIBLIOGRAPHICAL NOTICES.

*The South-Western Medical Advocate.* Edited by JAMES CONQUEST CROSS, M. D., Professor of the Institutes of Medicine and Medical Jurisprudence in the Memphis Medical College, assisted by his colleagues. No. 1. Vol. 1, for July 1847. Memphis, Tennessee.

It has ever been our wont to herald every new labourer in the arduous, responsible, and, too often unrequited and thankless field of medical journalism; and with sincerity have we welcomed them when ushered into existence under the banner of chivalry and honour, even although they may have attached themselves to some particular school or clique, whose interests they professedly watched over. The number of the Journal—if so it may be called—before us, by its very title indicates that it is not free from such bias; nor do we object to it on this account. To be the organ of the profession in the south-western portion of this extensive country would be an enviable position for the editor; but we feel assured, that if the forthcoming numbers are to be judged of by the present, the high-minded and liberal practitioners of that region will eschew it, and deny that the unqualified and reckless assertions contained in it are participated in by any one of them. The advocacy of south-western medicine is, however, but an infinitesimal portion of its objects. The grand *desideratum* is "to establish a medium of communication between the *Memphis Medical College,*" [an institution established immediately after the editor left the Transylvania school]—" and the *Medical Public* of the south-west;" and a secondary—perhaps primary—object would seem to be, to enable the editor to rail against all who have in any manner interfered with his devious course, and especially against his former colleagues of the University of Transylvania, who, some time ago, published, under their own signatures, statements, which it would be difficult to believe were without foundation, and from which, unquestionably, it has not been an easy matter for the editor to exonerate himself. Of this controversy, notwithstanding the number of missives that have reached us, we have hitherto expressed no opinion. For the sake of the individual most deeply concerned, as well as of that of the profession to which he belongs, and of the respectable school of which all the controversialists had formed part, we were silent: and we had hoped, that some discretion would have been exercised, and that if it had been deemed proper that such a discourse as the one that *fills* the first number of the "*South-Western Medical Advocate*" should be *delivered,* it most assuredly would never be *published,*—even although requested by a Board of Trustees, and by a medical—always indulgent—class.

The number before us is truly a "psychological phenomenon." It is *wholly* occupied with "*An inaugural discourse on the policy of establishing a school of medicine in the city of Memphis, Tennessee.*" It is essentially the editor and the Memphis Medical School—*Ego et rex meus !*—A goodly portion treats of the editor ; another portion of the Memphis Medical School; whilst no little space is devoted to the threadbare arguments on "sectional medicine," and to show, that a south-western physician must be educated in the south-west,—of course at Memphis, upon the same principle, we presume, as the old Joe Miller belief, that if a man be born in a stable he must necessarily be a horse;—that northern schools are inadequate to teach southern students, —that northern pathology and therapeutics are not southern pathology and therapeutics;—and farther, that the respectable gentlemen who fill so well the different chairs in Louisville, and in Lexington,—where the editor himself taught so lately, and for which he was so long the energetic advocate,—are wholly unfit to teach those who are destined to be south-western practitioners. What will the veteran professor of the former school—who has been so long the ardent advocate of "sectional medicine,"—as it has been termed—say to this? when he finds his own arguments returned to him, and that he and his colleagues are deemed as unfit to prepare students for the south and southwest, as we of the north are?

But all these views, and the establishment of the Memphis Medical school itself, are—it would seem—the suggestions of the purest philanthropy, untainted by the slightest selfish motive!

"Is another school of medicine necessary? This we have alleged; but an unsupported and independent assertion upon so important a subject, would be justly treated with ridicule and contempt, and unless we shall be able to appeal to satisfactory reasons, flowing from public necessity, there will be ground to suspect, if not to believe, that the individual who now addresses you, as well as those with whom he is associated, have been induced to embark in the enterprise from exclusively selfish considerations. Than this, we are sure, no more unjust or injurious imputation could be cast, at least upon our colleagues." [A wise reservation!] "We hope, therefore, to receive a patient, if not an indulgent hearing, while an attempt is made to recount to you some of the leading considerations that have caused the present enterprise to be undertaken." p. 3.

One of these "leading considerations," to which the editor repeatedly refers, is, that "in the schools of the north, as well as in those of Kentucky and Ohio, not a single teacher is to be found, who, from personal observation and experience, is qualified to impart a correct knowledge of the nature and treatment of the diseases that prevail in the South. This fact, which is utterly incontestable, has failed to attract the attention to which it is entitled, and although there may be those disposed to overlook or underprize it, in my humble judgment, it is of very great if not paramount importance." p. 5.

And are these the views the editor has always maintained? We suspect not;—for we have a faint recollection of his having visited the northern cities, some years ago, for the purpose of procuring a Professor of the Principles and *Practice* of Medicine, for the medical department of the University of Transylvania, when he most strenuously but unsuccessfully exerted himself there for that purpose. He may reply, indeed, as did another professor of the west, on a different occasion, that he was then acting for his colleagues, not for himself. The professor alluded to had been a zealous teacher in a town of not more than eight thousand inhabitants, which *then* he thought, or maintained, possessed the necessary facilities for anatomical and clinical instruction. On being translated, however, to a town of thirty thousand inhabitants—*cœlum* et *animum mutat*—he now finds that the former position was totally devoid of the requisite facilities ; and loudly proclaims the superior advantages, in these respects, of his new position. On being asked how he reconciled his *past* and his *present* views : he replied, that in the former case he was but expressing the views of his colleagues, in the latter his own !

The editor may say, that his agency for the Transylvania school, and the arguments which he adduced in its favour, were but the delegated views of others; but he certainly succeeded in impressing those with whom he came in contact, that they were as strongly his own.

But when did he first learn, that the schools of Kentucky and Ohio are liable to these same objections that have been imagined to apply to the northern ? Only, we apprehend, since he quitted so summarily the one to which he was attached, and to which he alludes so bitterly in many parts of his "Discourse." He suffers, indeed, no opportunity to pass by for vilifying it, or some of its able teachers. Thus, when referring, in a note, to Dr. Condie's opinion, that the immense doses of sulphate of quinia given by many of the southern physicians are "certainly excessive and uncalled for," he exclaims :

"How does he [Dr. Condie] know ? Has he ever seen a case of congestive remittent fever in the South ? No. Then he knows nothing about it, and has no right to question the skill and judgment of those who are familiar with the subject. This is like the philippic which Dr. Dudley is in the annual habit of pronouncing against quinine and iodine ; and yet he has the assurance to tell his classes he never employed them in his life." p. 6.

And again:

"The diseases of the northern part of Kentucky, and of the whole of Ohio, differ in several striking respects, from those that are observed in lower latitudes: those, therefore, who undertake to teach medicine in either Louisville, Cincinnati, or Lexington, without having previously acquired a personal knowledge of those of the latter, will never, so long as they remain in those cities, become competent teachers of those who intend practising the profession in the South.

And again:

"But the alumni of the Atlantic schools, as well as those of Kentucky and Ohio, regardless of the great fundamental facts that the pathological phenomena and therapeutical indications of the maladies of the South differ widely from those that are observed in such as occur independently of intense heat and a malarious atmosphere, and believing they have been taught correctly, without hesitation or remorse, subject the southern constitution to rules of treatment that are no less absurd in principle, than they are frightfully fatal in practice. We feel authorized in asserting, as the result of the multiplied experience and observation of all ages in low latitudes, that the liver is the great emporium of both health and disease in hot climates. In all its pathological and therapeutical relations it is an organ of paramount importance to the practitioner, for it is, as we have said, directly or indirectly concerned in most of the modes of morbid action which prevail in the South. A thorough acquaintance with the physiology, pathology, and therapeutics of the great organ, so far as understood, is therefore entirely indispensable to the successful treatment of Southern maladies."

And he observes subsequently and characteristically:

"The liver, whatever may be said by those, who, in reality, know nothing on the subject, but who, from motives perfectly intelligible, may be disposed to set aside or obscure the perception of the truth, is as much of a pathological autocrat in the South, as the lungs are in Philadelphia or Boston—in Louisville, Cincinnati or Lexington; and the other organs of the body are as completely under the presidency of the former in the South, as they are under that of the latter at the North."

All this, however, is mere twaddle, conveyed, it is true, in a sort of professional language, but not the less twaddle. It is the view which led calomel to be considered in the West, South, and South-west as the "Samson article of the Materia Medica," a Samson whose strength, however, is now gone from it; and this result brought about, we believe, in part, by the teaching of certain northern professors; but assuredly it is not the view under which the sulphate of quinia is now regarded as the southern "Samson!" And can we, in charity, believe, that the Editor has forgotten that one of the most powerful advocates for the *febrifuge* virtues of this valuable agent is Professor Mitchell, of Lexington,—a teacher in one of the very schools, which, according to him, "are, no matter what ingenious sophistry may be employed to prove the contrary, necessarily disqualified to impart such information as will prepare their Alumni to encounter with success the mortal endemics that annually desolate those regions."

And, then, to prove, that of "the practice so common and firmly established in the South-west of arresting, or jugulating, as the French term it, remittent fever," the teachers and writers of the Atlantic States and of the West are "entirely ignorant," the Editor quotes an article by Dr. Ford of Georgia, published in the Georgia Medical Journal, in which that gentleman refers to different "Practices of Physic" published in the United States; but to do their authors justice—one of them at least—it would have been but proper to refer to the latest edition, and to a part in which the quinia treatment *is* referred to. It is too much, however, to expect that authors—even so *distinguished* as he of the "Chronothermal System," whom we find the Editor citing as *authority* in more than one part of his "discourse"—can keep up with sudden *pirouettes* in practice, and a more signal one certainly never occurred than in the transition from the calomel to the quinia management of southern fevers. Some of the works cited were published years ago, and when the plans of treatment were by no means as developed and as general as they are at present.

And, what a gross and unfounded libel we have in the following assertion!

"The statistical results of Dance's experience, or rather experiments, as embodied in his *Treatise on Fevers,* are a melancholy comment on the curative efficacy of French medicine, of which North-eastern practice is almost a literal transcript, for medical expectation, which Asclepiades properly called a meditation on death, constitutes the sum and substance of the former, is the common and current practice of the latter. p. 21.

It appears, however, that epidemics differ as much as "sec tional" diseases; and therefore, we presume, we must have a special school for each epidemic!

"No physician has ever witnessed two epidemics of precisely the same character, that would yield to precisely the same treatment, or that would submit to the management followed with success in sporadic cases. There is a tendency to death in a particular way in each epidemic, and this the physician must discover, and if possible resist." p. 23.

And as if to "outherod Herod," we have the following assertion to which we shall not attach an epithet that any one at all acquainted with facts could readily supply.

"The inability of northern physicians, as well as those who reside in temperate latitudes, to treat successfully the maladies of the south-west, is conclusively proved by the enormous comparative mortality which is observed amongst those who, after having resided for months, in succession, in a malarious region, migrate to cold latitudes and subsequently sicken. From what we have actually observed" [in all the northern or middle states of the Union, we presume!] "we do not hesitate to affirm that it is almost equivalent to a sentence of death for south-western men to sicken of any acute disease, and especially of fever, in any of the northern or middle states of this Union."

*Quis credat? Non ego!* Yet the editor says it is so: "and sure he is an honorable man"!

Again:

"It has been alleged in this discourse that a great degree of scepticism in relation to the curative powers of therapeutical agents prevails amongst the dignitaries of the profession at the north, and we may say from personal intercourse with medical men of the highest respectability, and without injustice to them, that it exists to almost an equal extent in the larger cities of the west. Nothing of this sort is observed at the south-west."

All which is, of course, intended to show the superiority in *practical* knowledge of the south-western physicians.

The Editor now no longer pipes: he actually "bursts the bag"!

"In the south-west, neither the homœopathist nor the anatomo-pathologist will find countenance or encouragement. Disease is much too violent and malignant, and the efficacy of active and energetic treatment has been too often witnessed, is too well understood, and is too justly appreciated for either of them to be able to take root or to flourish. This is impossible, unless the homœopathist practises secretly what he has not courage or honesty enough openly to avow, and this it is believed he does in all latitudes; or the anatomo-pathologist abandons the anatomical basis of treatment, and endeavours to relieve human suffering by studying and analyzing symptoms, and observing the effects of remedial agents. Since, therefore, the public in the north-eastern portions of the United States has been reduced to the desperate necessity of selecting between homœopathy and medical expectation, and as, from a full trial of both, it has been constrained to decide in favour of the former, it must appear to every intelligent and reflecting individual, at all acquainted with the complaints that result from malaria or intense heat" [neither of which is known, of course, in the north!] "the most preposterous of all absurd ideas, to send young men to the north-east or west, in order to prepare them for the practical duties of the profession in the south-west." p. 36.

We have before adverted to the fact, that the editor was a strenuous advocate, at one time, for the appointment of a north-eastern teacher to the chair of *Practice* of Medicine in the Transylvania Medical School. It now appears that such professors, and all western professors, are not only absolutely unfit, but must forever remain unfit for any such office in the south-western schools. Speaking more especially of Louisville, Cincinnati, and Lexington, he says:—

"In neither of them can a teacher be found by whom the pathological and therapeutical principles, on which the treatment of south-western maladies is regulated, are taught. This every sensible man must regard as a great defect in the organization of the faculties of these institutions, for at least one-half their alumni are destined to find homes and to seek their fortunes in the lower latitudes of the Union. Nor is this all, or the most mortifying or discouraging part of the truth on this subject. Whenever an occasion has arisen which put it in their power to reflect honor and distinction by promotion to the dignity of a professorship, they have almost invariably preferred north-eastern men, who know nothing of western, and especially of south-western maladies. That such persons are unfit for the functions they are expected to perform, we have already shown; that they will always remain so we assert from personal observation, as we know it to be impossible to divorce the confirmed and committed anatomo-pathologist from his 'great pathologica and therapeutical principles.'" p. 37.

And moreover:

"The reasons why they" [south-western physicians] "have not been permitted to take a more distinguished part in the teaching of medicine in the United States, are obvious and intelligible. They observe and reflect more but write less than the physicians of the large cities of this Union; because what they do write" [the editor for example !] "is for the instruction of the profession, and not to sustain some 'great pathological principle of universal application;' because they aim to become practically useful as practitioners, and, if need be, teachers, and not to win conspicuous stations by becoming voluminous compilers of the results of other men's experience; because they have less book but more clinical knowledge; and finally, because the trade of criticism is exercised by north-eastern men almost exclusively. The result is, north-eastern books and north-eastern men are praised with little reference to their intrinsic merits, while south-western books and south-western men are overlooked and neglected or condemned." (?) p. 38.

In no way can this altogether supposititious evil—a creation of the author's own prolific brain—fearfully prolific in such conceits—be successfully rectified, he thinks, except "through their own schools and journals of medicine—through the instrumentality of which the ablest of themselves" [iterum ego !] "are the teachers, and in publications for which the most industrious, observing and talented of them are the writers." p. 39.

But the profession of the south-west require no such absurd vindication. We can affirm, most positively, that the kindest and most liberal sentiments are entertained towards them in this portion of the Union: it would be ridiculous to suppose that it could be otherwise; but if any thing were calculated to break in upon this most desirable harmony of feeling, it would be such loose, gratuitous, and indiscreet statements as those we have just quoted. We do not believe that they will have such an unfortunate effect upon a solitary 'north-eastern' individual, but we will not say what estimate they may cause to be put upon the head and heart of him from whom they emanated. In our opinion they certainly are not creditable to either the one or the other. It is idle, too, for the editor to refer to the difference which he fancies to exist between the physicians of the north-eastern cities, and those of the south-west, in the former writing more, but thinking less than the latter. He himself is, perhaps, the most voluminous medical pamphleteer that the whole Union could furnish; and as for the amount of thought—well directed and useful thought—contained in his pamphlets, all we shall say is, that the extracts we have given are amongst the *most favorable* specimens of his various polemical sheets, and fragments of sheets, which have been inflicted upon us. Of the gross illiberality that pervades the "Discourse," almost every page furnishes one or more examples, and where the object is to vent illiberal feeling, the language certainly savours, at times, too strongly of hyperbole. Thus, in speaking of the school, once highest in his favour—the one at Lexington—the south-western neophyte of one year's standing remarks:

"Lexington, which does not contain over eight thousand inhabitants, had for many the second, and for some years the third medical institution in the United States, although, at present, from causes to which it is now useless to advert, it has sunk so low as to be, we believe, the tenth or twelfth of the Union.

Where is the authority for this? *We* know of none.

It will be observed, that we have entered into no vindication of the north-eastern schools against the attacks made upon them by the editor of the *South-western Medical Advocate*. It would be idle to answer that which requires no answer, and the object of which must be apparent to the most careless reader. We have been desirous only of shewing up the Journal and the Editor by his own shewing; to announce, in sporting language, "the name of the horse and the colour of the rider;" and the result must have been a desire on the part of every friend of the whole profession, and especially of south-western medicine, that this South-western medical *Detractor,* rather than South-western medical *Advocate,* should quit the course—that it should cease where it has begun, and that the prolific author should follow the example set him by Cid Hamet Benengeli, in the last chapter of Don Quixotte, and promise repose to his pen. Especially would we advise him—although we are satisfied that the office of adviser is, at best, but a thankless one, and that the advice will be wholly thrown away—to be a shining specimen, not of the *Acarus Crossii,* which has so much puzzled the naturalist, and whose precise position it has been so difficult to define, but of the south-western genus of physicians, as he describes them, "who observe and reflect more, but write less than the physicians of the large cities of this Union, because what they write is for the instruction of the profession." Let him but succeed in this, and we shall be the first to herald and applaud the happy change.

---

*A Treatise on the Practice of Medicine.* By GEORGE B. WOOD, M. D., Professor of Materia Medica and Pharmacy in the University of Pennsylvania; one of the Physicians to the Pennsylvania Hospital; one of the Authors of the Dispensatory of the United States of America, etc., etc. 2 vols. 8vo. Grigg, Elliott & Co. Philadelphia, 1847.

Formerly, the appearance of a general Treatise on the Practice of Medicine was the event of an age; of late years, it is scarcely less than an annual occurrence. So numerous are they on our shelves, and some of them so complete, that one is at a loss to conceive of the occasion for a new one. Until lapse of time and the advancement of our science have added new facts, or shed more light upon what is already known, it would seem difficult for a writer to find materials out of which to construct such a work, without injustice to his readers, and to those from whom he derives whatever is valuable in his production.

Dr. Wood experienced the truth of these observations, and accordingly, in his preface we find the following apology.

"In adding another to the many valuable Treatises on the Practice of Medicine, the author may be reasonably expected to show, upon what grounds he has ventured to advance a new claim to the public attention, already so fully occupied. He has no other excuse to offer than this; that he has written in obedience to impulses which he could not well resist. Having been engaged, for nearly thirty years, in public and private practice, and, during that time, devoted an almost exclusive attention to the study of diseases and their remedies, he has accumulated facts, and formed opinions, which have been long soliciting expression, with an urgency to which he has at length yielded, though unfeignedly distrustful of their sufficient value."

What are the facts which have been accumulated, and whether derived from authors who have preceded him, or exclusively of his own discovering, and in relation to what subjects, is not stated in the preface, and consequently it is only after tracing them out by searching through the work, that we are enabled to appreciate them. From the connection in which the subject is presented, we infer that the facts alluded to are original with the author, although not so clearly stated, and if so, it is to be regretted, considering how few are the discoveries of this kind which it is the fortune of a single observer to bring to light, that time and further investigations confirm,—it is to be regretted, we say, that these, which ought to be the gems of the work, were not more specifically pointed out: it would at least have saved the reader the task of wading through two ponderous volumes to find them. The "Opinions" which the author, in the course of "nearly thirty years, in public and private practice," has formed, are neither so sparse nor so difficult to discover, and, after all, constitute the most interesting as well as original part of the work. Without dogmatism of expression, they are yet put forth on most occasions in a way that leaves little room to suppose that any doubts are felt of their correctness. When reference is made to the opinions of others, it is in a courteous spirit, and rarely for the purpose of criticism. On other occasions, dissent and commendation are equally withheld.

Dr. Wood has divided his treatise into *two Parts*, each divided again into *sections*, *sub-sections*, and *articles*.

Part I. is devoted to General Pathology and Therapeutics, and consists of four chapters; which treat respectively of the "constituent forms of disease, as disease of the fluids and disease of the solids; causes of disease, or etiology; symptomatology, or semeiology; course, duration, and termination of disease; diagnosis; prognosis; general therapeutics, embracing *general indications* and general *therapeutic processes*.

Part II. treats of special pathology and therapeutics, and is divided into *three classes*, under which are arranged *general diseases*, *constitutional diseases* and *local diseases*. Under the head of *general diseases* we have fevers—including plague, variola, vaccina, varicella, rubeola, scarlatina, and erysipelas; the class of *constitutional diseases* includes only *rheumatism* and *gout*; whilst that of *local diseases*, amongst a vast catalogue, embraces *insanity* and *delirium tremens*—*epilepsy* and *chorea*, *hydrophobia*, *hysteria*, etc.

In the yet unsettled state of pathology, all classification of diseases must be imperfect, and consequently arbitrary, but it seems to us that this one does not represent the present state of our knowledge. Take, for example, the two last named diseases, and apply the author's own definition of the terms, and see how far they answer to the character of local diseases. " Hydrophobia is a peculiar disease, resulting from the entrance into the system of the poison of a rabid animal." It is not alledged that the local phenomena that are described, " such as aching, tingling, burning, coldness, numbness, or stiffness in the cicatrix" constitute the disease, any more than the pustule which follows the insertion of the virus of small pox or vaccine into the arm. In the former as in the latter instances, the disease is referred to "*the entrance into the system of the poison,*" and why in the one case it should be regarded as a *local* and in the other as a *constitutional* disease, it is difficult to conceive. Again: "Hysteria is a disease consisting in a morbid excitability of *the general nervous system,* [the italics are our own,] showing itself in occasional convulsive paroxysms, and diversified functional disorder." As already remarked, all classifications of diseases are necessarily to some extent arbitrary ; an author may therefore devise or adopt that which may best accord with his own views, but it is not to be expected that he shall contradict himself—that the phenomena which constitute the disease, according to his own description, shall be *general,* whilst it is included in the catalogue of such as are regarded as *local.* It is not our purpose, however, to enter upon an extended criticism of the work, but, having mentioned its general contents, make such occasional comments as the nature of the subjects may suggest, and our restricted limits permit. In our " devious course" we shall doubtless find much to commend, and, perchance, something from which to dissent.

In his observations on general pathology, under the head of *Depression,* we find the following remarks, which are a fair specimen of the author's style and mode of reasoning:

" There are three conditions in which the system is below the ordinary standard of health, and which, though if slight and of brief duration, they would scarcely be accounted morbid, are decidedly so when considerable or protracted. These three conditions are often confounded under the name of debility, or asthenia, but are essentially distinct, and sometimes require different if not opposite modes of treatment. It is, therefore, important to have clear conceptions of their nature, and to be able practically to distinguish them. The conditions alluded to are depression, debility, and diminished excitability. It must be borne in mind that action, the power to act, and the susceptibility to the influence of excitant agents, which is here denominated excitability, are different conditions or qualities of the system, and may each be reduced without a necessary reduction of the others. A morbid diminution of action is *depression*, a similar diminution of power is *debility*, and the term *diminished excitability* explains itself."

"These conditions," the author remarks, "may and often do co-exist;" but he thinks they are essentially distinct, and may exist separately. This position he explains as follows:

"That power may be diminished, without a diminution of excitability or of action, is no less true. It is notorious, that a debilitated system is often thrown into tumultuous disorder by causes which would produce no such effect in health. Thus, in an individual much reduced by an impoverished diet, or by depletion, how often do we observe a panting respiration, and an almost convulsive action of the heart, under a degree of bodily exertion which would have scarcely discomposed these functions, in the ordinary state of the system! How often, under similar circumstances, do the slightest causes produce great nervous disturbance, amounting in the female to violent hysteria! These results can be accounted for only by admitting an increased excitability, which enables the same amount of excitant influence to produce a greater amount of effect."

Dr. Wood may be regarded as a strenuous advocate of the modern doctrine of humoral pathology. He considers it "certain that, in some diseases, as in malignant fevers, for example, the blood does undergo great deterioration, either from the direct action of the poison introduced, or from derangement of the organs, and thus becomes wholly unfit to support the healthy actions, which are consequently greatly depressed."

We are glad to find the following sound views expressed on a subject too much misunderstood by many physicians, who seem to think that excitement is always to be overcome by means that reduce the strength of the system, and especially by blood-letting.

"But it must be borne in mind, that a mere loss of blood, or a mere diminution of its stimulant properties, without any positive noxious deteioration, though it may cause debility, does not necessarily diminish excitability. On the contrary, the organs feel more acutely the impression of other excitants, and are stimulated into excessive action by causes which would not have this effect in health. I have before alluded to the tumultuous disorder produced under these circumstances, in the respiratory and circulatory systems, by moderate muscular exertion, and in the nervous system by slight causes of disturbance. Even without any apparent excitement, the heart very often acts with greatly increased frequency. This phenomenon, however, is susceptible of explanation. The nutrition, muscular contraction, digestion, and other functions constantly going on in the system, require a certain amount of blood for their support; and in order to insure the requisite supply, nature has established such a sympathy between the parts in which these functions are performed, and the heart, as to call the latter organ into increased action, when any deficiency is experienced." (page 62.)

Whatever may be thought of the explanation, the practical deduction is of very great importance in the treatment of disease. In an enfeebled condition of the system, to bleed, purge and starve, because the heart acts tumultuously, is literally to slay the patient; and yet, regardless of all the other indications, we have known extensive bleeding performed only a few hours before death, *because the heart was beating with unwonted force!* In such cases, it will always be found that instead of being curbed, the *frequency*, at least, of the heart's contractions will be increased, and the danger vastly augmented.

The author treats of *revulsion, derivation*, and *counter-irritation*, as one and the same thing—as if, in fact, these were convertible terms. Thus he remarks: "This [revulsion—derivation—counter-irritation] consists in the diversion of disease from one part of the system, by the production of inflammation, or irritation, in another part." (p. 216.) This is true of counter-irritation, but very effectual revulsion is often made by means which excite neither irritation nor inflammation. The various hygienic influences so successfully invoked in the treatment of many chronic diseases act by the production of healthy excitement of the various organs of the body, without that excitement rising to the degree of irritation.

After explaining the action of revulsive agents (or rather of counter-irritants) the reader is very properly cautioned against their employment in the active stage of inflammatory diseases.

"But," observes the author, "during the existence of high constitutional excitement, the revulsive agent is not able to unseat the inflammation, and, if of itself irritant, as in the instances of rubefacients and blisters, may add to the existing excitement, by the sympathy of the system with the superficial inflammation they produce. But, when the revulsive impression is conjoined with copious depletion, as in the case of hydragogue cathartics, which produce a revulsion towards the whole lining membrane of the bowels, while they evacuate the contents of the blood vessels, it may be resorted to even in the greatest height of the inflammation. The copious secretion prevents the excitement of an irritation in the bowels, sufficient to bring the constitution into sympathy." (p. 217.)

We cannot assent to the correctness of the practice inculcated in this paragraph. Although hydragogue cathartics produce "copious depletion," it is only of the watery part of the blood, and not its richer and more stimulating portion ; and beside, the action of several medicines of this class is sufficiently energetic to cause so great local excitement as to materially effect the general nervous and vascular systems. We think that Dr. Wood himself, on reflection, would hardly prescribe colocynth, scammony or gamboge in hepatitis, peritonitis, or pneumonia, "in the greatest height of the inflammation ;" trusting to the copiousness of the secretions to counteract their general excitant influences.

We have noticed occasional instances like these in which we differ from the author in points of practice, and in which we think he differs from *himself*—for in the general soundness of his therapeutical principles we entirely concur. They are such as we have acted on for a third of a century, slightly modified, it may be, by the progress of our science. It is, however, in carrying into practice these principles, that we should have occasion to differ from the author, and on a closer examination of the work, it is not unlikely that these occasions would be multiplied. We perceive, for instance, the greatest confidence reposed in certain articles of the materia medica, to which we attach very little ; such as the *citrate of potassa* in fevers of almost every type, but especially bilious fevers. The acetic and tartaric acids in combination with potassa, according to Dr. Wood, will not do—nor the citric acid with either of the carbonates of soda. "I feel very confident," he remarks, "that no practitioner who has ever been in the habit of using the effervescing draught, *properly pre-*

*pared,* (i. e. fresh lemon juice and carbonate of potassa, in the effervescing state) in the treatment of bilious fever, will be disposed to abandon it." We have no doubt that this is true, and that those who are "in the habit of using" any other article will not be disposed to abandon it. "Habit" is a strong law, that few venture to dispute. We acquire the "habit" of giving certain things in particular diseases, and, in self-limited diseases, as fevers often are, our patients generally get well, and of course the medicine, no matter how inert, gets the credit of the cure, and our "habit" is confirmed.

We do not object to the efferverscing draught in bilious fever. It is as simple and innoxious as any thing else, and if something must be done to keep the patient and the doctor quiet, it may as well be given, although we should much prefer a piece of ice or a little cold water: but we object to overweening confidence in any single article in all cases of disease wearing the same general character, and especially to the ascription of very active properties to such as are really inert under almost any circumstances.

Generally speaking, the pathological principles, as well as the therapeutical laws, set forth in this treatise, will be found in accordance with the present advanced state of our knowledge on these subjects. Usually, it is not the author's custom to doubt, even on points regarded by the generality of authors as doubtful. Thus, yellow fever is neither gastritis nor a form of bilious fever, nor miasmatic, but a *peculiar* fever.

"There can, I think, be no doubt, that yellow fever is an entirely peculiar and distinct disease. What are the precise pathological conditions essential to it are unknown. It is highly probable that the blood is prominently in fault, being deranged by the immediate action of the morbific cause."—(p. 308.)

Congestive, or, as the author calls it, *pernicious* fever, is a "form of miasmatic fever," in which the congested condition of the vessels of certain organs is merely a consequence of the disease, and "it is in the peculiar state of the innervation, that we are to look for the source at once of the symptoms and the danger."

Continued or Typhoid fever he denominates "*enteric fever,*" and makes it, in its causes, symptoms and character, a distinct disease from *typhus,* thus cutting the knotty question of identity in the most summary manner, although M. M. Louis, Gaultier de Claubry and others, believe their typhoid and typhus to be absolutely identical : and one of the most recent numbers of the Archives Generale des Mèdicine (for May, 1847,) states that typhoid fever is one of the most irritating questions to the Academy de Medicine, and one on which the opinions of its members are most divided and most in contradiction. Its identity with remittent or bilious fever is also denied, notwithstanding the author admits that "cases having all the essential characters of enteric fever occasionally end in intermittent; and bilious fevers, or affections which cannot be distinguished from them, sometimes show signs of enteric fever during their progress."

The "present work claims to be something more than a mere compilation." The author does not, however, pretend that the facts or opinions advanced are generally original with himself: but that "these materials have for the most part been maturely considered, have been submitted to the closest scrutiny of which he was capable, and have been re-arranged in accordance with his own best judgment." This is certainly the true course for a writer to pursue who desires to prepare a comprehensive treatise which shall do full justice to the subject without undue prolixity. To do justice, however, at the same time, to antecedent and cotemporary writers and observers, by awarding to each the credit of what properly belongs to him, when thus "re-arranging" these materials to suit an author's own judgment, is not so easy a matter; and we regret to find that in the work before us the effort does not seem to have been always made, where early impressions or personal predilections were not strong enough to incite to the task. We think there are many instances overlooked where the writings of American physicians might have been profitably cited, whilst the published opinions of some of the most eminent of the profession, whose names are rarely if at all alluded to in the course of the work, would have given additional interest to its pages.

We have thus cursorily noticed some of the points on which we do not entirely accord with the author in the work before us. Candor would not allow us to do less. We are fully sensible of the difficulty there is in speaking of the productions of a writer whom we have known and esteemed through many long years of uninterrupted friendship, but we have endeavoured to do so with the impartiality demanded by truth and science, and it is in this spirit that we pronounce the treatise of Dr. Wood, notwithstanding the objections we have made, to be one of the most valuable of the works that have come from the American press, and exceedingly creditable to the zeal and abilities of the accomplished author.

---

*Elements of Chemistry, including the history of the imponderables, and the Inorganic Chemistry of the late Edward Turner, M. D., F. R. S. L. and E., Seventh Edition, and the Outlines of Organic Chemistry, by William Gregory, M. D., &c., Professor of Chemistry, University of Edinburg. With additions*, by James B. Rogers, M. D., Professor of General Chemistry, Franklin Institute, and Lecturer on Medical Chemistry, &c., and Robert E. Rogers, M. D., &c., Professor of Chemistry and Materia Medica, University of Virginia, &c., Svo. pp. 848. Thomas, Cowperthwaite & Co., Philadelphia, 1846.

Dr. Turner's elements of Chemistry has for many years sustained the highest character, both in Europe and America, as a full and accurate exposition of the principles of the science. It has already passed through several editions in this country, under the careful supervision of Professor Bache, to whom the Author, in the European edition, published shortly before his death, acknowledged himself indebted for the correction of numerous errors and the addition of much useful matter. We are pleased to find that the editors of the present edition have adopted these corrections of Dr. Bache, to whose labours they refer in the following courteous and just language: "In labouring to correct the numerous typographical errors of the London work, the editors have been greatly assisted by the American re-prints of the former edition of Turner's Elements, the great accuracy of which reflects so much credit on the industry and attainments of their accomplished editor."

In noticing a work which has passed through so many editions, neither analysis, criticism, nor commendation, is required or will be expected. All that seems necessary is to inform the reader of the changes and additions contained in the edition before us. These are well set forth in the following extract from the preface.

"The present blended republication embraces the whole of the Imponderables and Inorganic Chemistry of Dr. Turner's well known Elements, together with the Organic Chemistry of Professor Gregory's Outlines, and the separate treatises on Mineral and Organic Analysis by Mr. Parnell and Professor Liebig. In the first plan of the work it was proposed simply to re-edit the seventh edition of Turner's Elements, making such additions and emendations as the progress of the science required, and at the same time re-moulding the part devoted to Organic Chemistry in an abridged and systematic shape. The opportune appearance of Professor Gregory's Outlines greatly aided the accomplishment of this plan by furnishing ready prepared, just such a condensed and methodistical treatise on Organic Chemistry as was originally proposed, and the publishers availing themselves of so valuable an aid, at once determined upon substituting the second parts of the outlines in place of the intended abridgement of the Organic Chemistry of Dr. Turner's work. Of the propriety and utility of this change, the editors believe no doubt will be entertained, when it is remembered that the outlines are from the same pen that drew up a large part of the Organic Chemistry in the seventh edition of Turner; that they are in reality compiled of the same materials, with the addition of some more recent results, all very skilfully recast, and that they conform in notation and general method with the inorganic portion of Dr. Turner's work.

In the department of Analytical Chemistry, the closing division of the work, the editors conceive that the present publication will be admitted to present a very important improvement. This portion of the elements though enriched in the seventh edition by an able sketch of Inorganic Analysis, written expressly for it by Mr. Parnell, contains no account of the methods of *Organic Analysis*, a subject of peculiar and perhaps leading interest in its connection with modern chemical research. To remedy this defect the editors have gladly introduced Liebig's outline of the processes for analysing organic bodies, a treatise of the highest authority with all who are engaged in this department of science, and one happily adapted to the wants of the practical student."

The union of these several treatises constitutes the most comprehensive work, as to facts and principles, and the processes and applications of the science of Chemistry, to be found in the

English language. The task of the editors, it appears to us, has been well and ably performed, and we can most conscientiously recommend the present addition of Turner's Chemistry as the best adapted for the thorough study of the science of any with which we are acquainted.

---

*Braithwaite's Retrospect of Practical Medicine and Surgery.* No. XV., January to July, 1847.

We have received both the English and American editions of this useful publication. The "Uniform American Edition," which is a reprint of the English, is published by Daniel Adee, of New York, and the number before us contains 371 pages, octavo, of well selected matter. We very much wish that the American publisher would emulate the English in the mechanical execution of the work. The great efforts to sell American reprints at a very small price, which causes them to be driven through the press without being supervised by a qualified proof reader, and sent forth imperfectly printed and badly got up in all other respects, detracts very materially from their value. The constant misprints, especially in statistical tables, destroys all confidence, and, in fact, renders them quite valueless. This cheap publishing may do for novels and other light reading, when slight alterations or omissions can be discovered by the context, but in works of science it is really a serious matter. Besides, books which are to go into a physician's library for future reading and reference, should be constituted of permanent materials, and we have no doubt that publishers would find it to their advantage to bring out their medical publications in a style more befitting their contents.

